# Consistency of children’s hemodynamic responses during spontaneous speech

**DOI:** 10.1117/1.NPh.9.1.015003

**Published:** 2022-02-18

**Authors:** Seth E. Tichenor, Bridget Walsh, Katelyn L. Gerwin, Fenghua Tian

**Affiliations:** a Duquesne University, Speech-Language Pathology, Pittsburgh, Pennsylvania, United States; b Michigan State University, Communicative Sciences and Disorders, East Lansing, Michigan, United States; c Masimo, Irvine, California, United States

**Keywords:** functional near-infrared spectroscopy, speech, language, children

## Abstract

**Significance:**

Hemodynamic responses (HRs) are typically averaged across experimental sessions based on the assumption that brain activation is consistent over multiple trials. This may not be a safe assumption, especially in pediatric populations, due to unaccounted effects of inattention, fatigue, or habituation.

**Aim:**

The purpose of this study was to quantify the consistency of the HR over speech and language brain regions during speech production in typically developing school-aged children.

**Approach:**

Brain activity over speech and language regions of interest (ROIs) was recorded with functional near-infrared spectroscopy during a picture description paradigm with 37 children (aged 7 to 12 years). We divided the 30 experimental trials, each 5 s long, into three segments of 10 trials each corresponding with early (trials 1 to 10), middle (trials 11 to 20), and late (trials 21 to 30) trials. We then compared oxygenated (HbO) and deoxygenated (HbR) hemoglobin concentrations averaged across each 10 trial segment to overall concentrations averaged across all 30 trials. We also compared differential hemoglobin (HbD) across ROIs.

**Results:**

HbO and HbR averaged across all experimental trials most strongly correlated with HbO and HbR from early trials. HbD values from channels over most speech and language regions did not appreciably change throughout the experimental session. The exception was HbD values from channels over the dorsal inferior frontal gyrus (dIFG). This region showed significantly higher activation over the left hemisphere during the first segment of the experiment.

**Conclusions:**

Our findings suggest that brain activity from speech and language ROIs was relatively consistent over the experimental session. The exception was increased activation of left dIFG during earlier experimental trials. We suggest that researchers critically evaluate the consistency of HRs from different brain regions to determine the reliability of HRs recorded during experimental sessions. This step is instrumental in ensuring that uncontrolled effects do not mask patterns of task-related activation.

## 1 Introduction

English-speaking adults produce on average 2 to 3 words per second in connected speech.^1^ Formulating relatively errorless speech is a skill that develops from infancy^2^^–^^6^ and relies on multiple hierarchically and parallelly distributed speech and language neural networks.^5^^,^^7^^–^^10^ First, a concept (lemma) is selected from a person’s lexicon from a field of competitors.^5^^,^^11^ Semantic, syntactic, morphological, and phonological information is then attached to the word form over the course of milliseconds.^5^ This planned word form is translated to the speech-motor system, which sends signals to the articulators for production.^8^^,^^9^ Investigations into the neurophysiology of speech production and language formulation over the past few decades have led to a deeper understanding of how language formulation and speech production processes interact and operate in daily communication [see Refs. 9, 12, and 13, for review]. Functional near-infrared spectroscopy (fNIRS) has been used increasingly in speech and language paradigms with children because it is less sensitive to speech-related motion artifacts than functional magnetic resonance imaging [see Refs. 14-16], and is a more child-friendly neuroimaging method.^17^^,^^18^ Specifically, fNIRS does not require time spent in an MRI scanner, which can be fear- or anxiety-inducing for some children.^19^ These benefits are of particular relevance for studying many developmental speech and language conditions [see Refs. 20 and 21, for review], including our previous work in the area of developmental stuttering.^16^^,^^17^

In our prior study, we recorded children’s (aged 7 to 11 years) hemodynamic responses (HR) while they completed a picture scene description task. We found canonical hemodynamic activation over the left dorsal inferior frontal gyrus (dIFG) and left premotor cortex (PMC) in controls, while children who stutter showed a distinctly different pattern—deactivation in these regions of interests (ROIs).^16^ These ROIs are critical for speech production and processing and are accounted for in prominent models.^8^^,^^22^ It is hypothesized that frequently produced articulatory programs stored in IFG and PMC project to primary motor cortex for execution. These well-learned articulatory programs are activated from language production networks. Thus, IFG and PMC have bidirectional neuronal connections to other neural regions, such as the superior temporal gyrus (STG) and the lateral parietal cortex.^9^

In many paradigms exploring speech production and language formulation, including our earlier study, participants are typically required to perform identical or highly similar speaking tasks multiple times over an experimental session (e.g., verbal fluency tasks, picture description, silent sentence reading) so that HRs recorded across trials can be averaged and compared across groups or individuals. These conventional methods, used for both behavioral and neurophysiological speech and language research, are predicated upon a critical assumption of block experimental designs—that the HR over speech and language areas does not systematically change across repeated experimental trials. The assumption is made that speakers engage speech and language networks in a consistent manner resulting in reliable HRs across multiple experimental trials. To our knowledge, however, there are no data on the consistency of the HR measured with fNIRS during speaking tasks across an experimental session. It may not be a safe assumption that brain regions are consistently engaged (indicated by consistent HRs) across an experimental session given that participants may become disinterested or fatigued,^23^^–^^26^ particularly in pediatric populations susceptible to such experimental effects. Moreover, there may be increased hemodynamic variability over speech and language ROIs in more ecologically valid speaking tasks (e.g., spontaneous speech generation as opposed to single word reading or picture description tasks) in which participants’ utterances are less controlled.^16^ Brain activity in speech and language regions may also change over time as participants become more efficient as they complete identical or similar speech and language tasks,^2^^,^^3^^,^^9^^,^^27^ further questioning the assumption that HR can be reliably elicited over ROIs across multiple trials. These unaccounted-for effects may lead to inaccurate conclusions about language formulation, speech production, or group differences in disordered populations when HRs are averaged across an experimental session.

Further specifying whether or how the HR changes over the course of an experimental paradigm in children may directly inform base knowledge of developmental processes related to speech production and language formulation, along with experimental design principles. Thus, the purpose of this study is to quantify whether or how brain activity over language and speech regions changes during a connected speech task (spontaneously generated speech to describe picture scenes) in typically developing children.

## 2 Methods

### 2.1 Participants

Participants in this study were 37 children (20 boys and 17 girls) aged 7 to 12 years (M=9.11; SD=1.54). Data from 16 of these children were included as age-matched controls for children who stutter reported in our earlier publication [see Ref. 16]. Data from the remaining 21 children have not been previously published. All children spoke North American English, passed a hearing screening, and demonstrated typical oral-motor functioning on a screening assessment.^28^ Children scored within normative limits on a speech and language screener [Clinical Evaluation of Language Fundamentals Screening Test (CELF-IV)^29^] and were confirmed to have typically developed speech and language skills by a certified speech-language pathologist (second author). The Handedness Questionnaire^30^ was used to establish right-handedness.

### 2.2 Tasks and Procedures

Tasks and procedures including the experimental protocol, fNIRS recording, and registration of optodes are detailed in our earlier publication [see Ref. 16]. Briefly, the experiment required children to describe aloud 30 picture scenes. Each slow event-related trial began with the presentation of a unique picture scene. After 2 s, a green circle with the word “go” appeared in a corner of the screen to cue the children to begin describing what they saw in each scene. The green circle stayed on the screen along with the picture scene while the children spoke for approximately 4 s [see, Ref. 16 for visualization of this paradigm in our previous published work]. This brief speaking interval is also similar to task durations for overt speech in other published work [e.g., Ref. 31]. After this 4-s interval of speech, a stop sign cued children to stop speaking. The stop sign was then followed by a jittered 10 to 12 s rest interval allowing the HR recovery.

Two experimenters were present for experimental sessions. The experimenter sitting next to the child made notes on significant arm or leg movements, yawns/coughs, periods of distraction, etc. The other experimenter monitored fNIRS signals and transcribed children’s utterances. We reviewed audio/video files offline to confirm and reject trials corrupted by motion artifacts and to confirm transcription accuracy. Trials were also rejected if the child did not respond verbally to the task, failed to produce more than three syllables in their utterance, responded before the green go circle was presented, or responded after the stop sign was presented. Trial rejection was infrequent since all children completed practice trials with feedback to ensure that they understood the task and could comply with the instructions.

### 2.3 fNIRS Recording and Processing

**Fig. 1:**
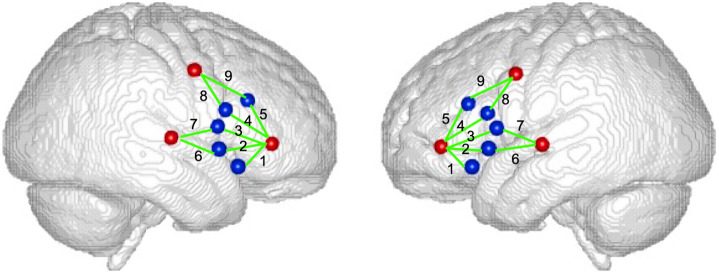
Left and right hemisphere probe. The brain templates, courtesy of John Richard’s lab, represent average whole-head MRI images (ICBM-152) from 72 10-year-old children.^32^ Left and right channels 1 to 3 recorded over vIFG, left and right channels 4 to 5 over dIFG, left and right channels 6 to 7 over STG, and left and right channels 8 to 9 over PMC.^16^^,^^17^

Children’s brain activity was recorded with a continuous-wave fNIRS system (TechEn Inc., Milford, MA) at a 50 Hz sampling rate, which used six emitting optodes at 690 and 830 nm as light sources and ten avalanche photodiodes as detector optodes to measure changes in diffused light. The probe was placed symmetrically over two hemispheres based on the 10–20 coordinate system [see Ref. 16]. The probe covered four ROIs in each hemisphere, as shown in Fig. 1. We targeted these ROIs because they are regions critical for speech encoding, translating articulatory motor plans to the primary orofacial motor cortex, and sensorimotor integration.^9^^,^^33^ The optode locations were recorded with a digitizing stylus (Polhemus; Colchester, VT) and later registered on a standard brain atlas taken from whole-head images of 72 children (aged 10 years through 10 years and 4 months) scanned by a 1.5T or 3.0T MRI (ICBM 152).

Recorded fNIRS data were preprocessed using Homer2 software.^34^ Briefly, raw data from each channel were visually inspected to ensure signal fidelity, i.e., that they showed a clear, real-time cardiac response to be included in analyses. Channel-wise signals were band-pass filtered (0.03 to 0.5 Hz). Hemoglobin concentration changes were calculated based on the Modified Beer-Lambert Law with a differential pathlength factor of 6.0.^35^ Our channel distance was 2.8 cm between each source and detector pair. Then, speech-related motion artifacts were removed from each channel using a correlation-based signal improvement (CBSI) approach^36^ that has been validated in previous studies.^17^^,^^37^ This approach is based on the fact that brain-related oxygenated hemoglobin (HbO) and deoxygenated hemoglobin (HbR) changes are negatively correlated, while motion-related HbO and HbR changes are positively correlated. Therefore, a real-time subtraction can be applied to remove the components of positive correction and maximize the components of negative correction.

Following CBSI, the data were downsampled to 2 Hz. To investigate the consistency of the HRs, channel-wise average concentration amplitudes of HbO, HbR, and differential hemoglobin (HbD = HbO – HbR) within a 3 to 8 s post-stimulus window were calculated for each trial, resulting in 37 participants × 18 (9 left and 9 right) channels × 30 experimental trials. The 3 to 8 s window was targeted to accommodate individual differences in participants’ hemodynamic peaks along with the temporal lag of the HR.^38^ Valid experimental trials were then divided into three chronological segments with the first 10 trials comprising the first/early segment, the second 10 trials comprising the second/middle segment, and the last 10 trials comprising the third/late segment. This division into thirds was chosen for two reasons. First, our paradigm contained 30 picture description trials, so dividing the data by early, middle, and late thirds ensured that each bin had approximately the same number of trials within. Second, many behavioral and neuroimaging investigations of speech and language processes in the field of communication science and disorders have used similar numbers of trials (i.e. ∼10) to determine effects [e.g., Refs. 31, and 39–42]. Thus, investigating the consistency of HRs over trial segments that included up to 10 trials was warranted.

### 2.4 Statistical Analyses

To determine the overall consistency of the HbO and HbR responses across the 30 trials, we compared the channel-wise HbO and HbR concentrations averaged across all 30 talk trials against the concentration values from each chronological segment comprising 10 trials (first/early, second/middle, and third/late) based on the Pearson’s correlation coefficients. Four analyses of variance (ANOVAs) with repeated measures on segments (first, second, and third) and hemisphere (left and right) for each of the ROIs—ventral IFG (vIFG), dIFG, STG, and PMC were conducted to assess potential differences in HRs with respect to segments and cortical locations. Given that HbO and HbR changes are negatively correlated post-CBSI, we used the differential hemoglobin signal (HbD = HbO – HbR) in these ANOVAs to decrease the number of statistical tests in evaluating whether ROI activation changed across segments. Topographic ROI images were created using a MATLAB-based optical toolbox^43^ based on the 10-year-old brain template^32^ and stereotaxic coordinates obtained from the registration procedure.

## 3 Results

### 3.1 Behavioral Results

Unusable trials, due to a participant failing to respond or significant head or body motion, for example, occurred infrequently. There was no difference in the number of valid trials available in each segment for analysis F(2,72)=0.53, p=0.59; Segment 1: M=9.62, SD=0.83; Segment 2: M=9.62, SD=0.68; Segment 3: M=9.76, SD=0.64. Because these movements were often unassociated with the experimental task, trials containing these were removed. Children also produced a comparable number of spoken syllables to describe the pictures scenes for each time segment: Segment 1: M=10.12, SD=3.02; Segment 2: M=10.32, SD=2.54; Segment 3: M=10.08, SD=2.88; there were no differences among the three segments with respect to the number of spoken syllables for each picture scene F(2,72)=0.66, p=0.52.

### 3.2 fNIRS Results

**Fig. 2:**
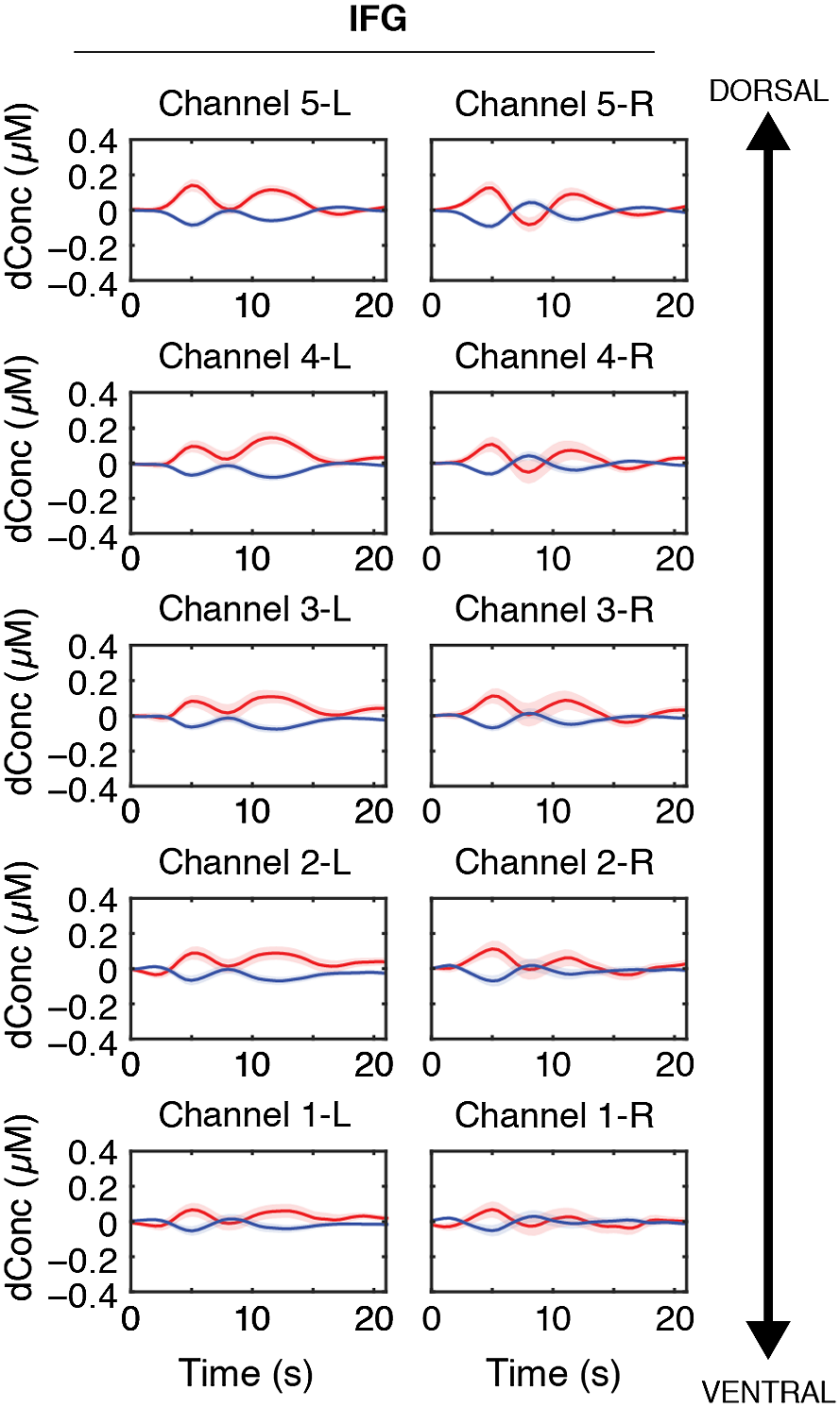
Grand averaged HbO (red) and HbR (blue) HRs with standard error curves are plotted by left (L) or right (R) ROI for the IFG. The IFG ROIs are arranged from ventral to dorsal anatomical locations. Time 0 indicates trial onset when pictures were presented.

**Fig. 3:**
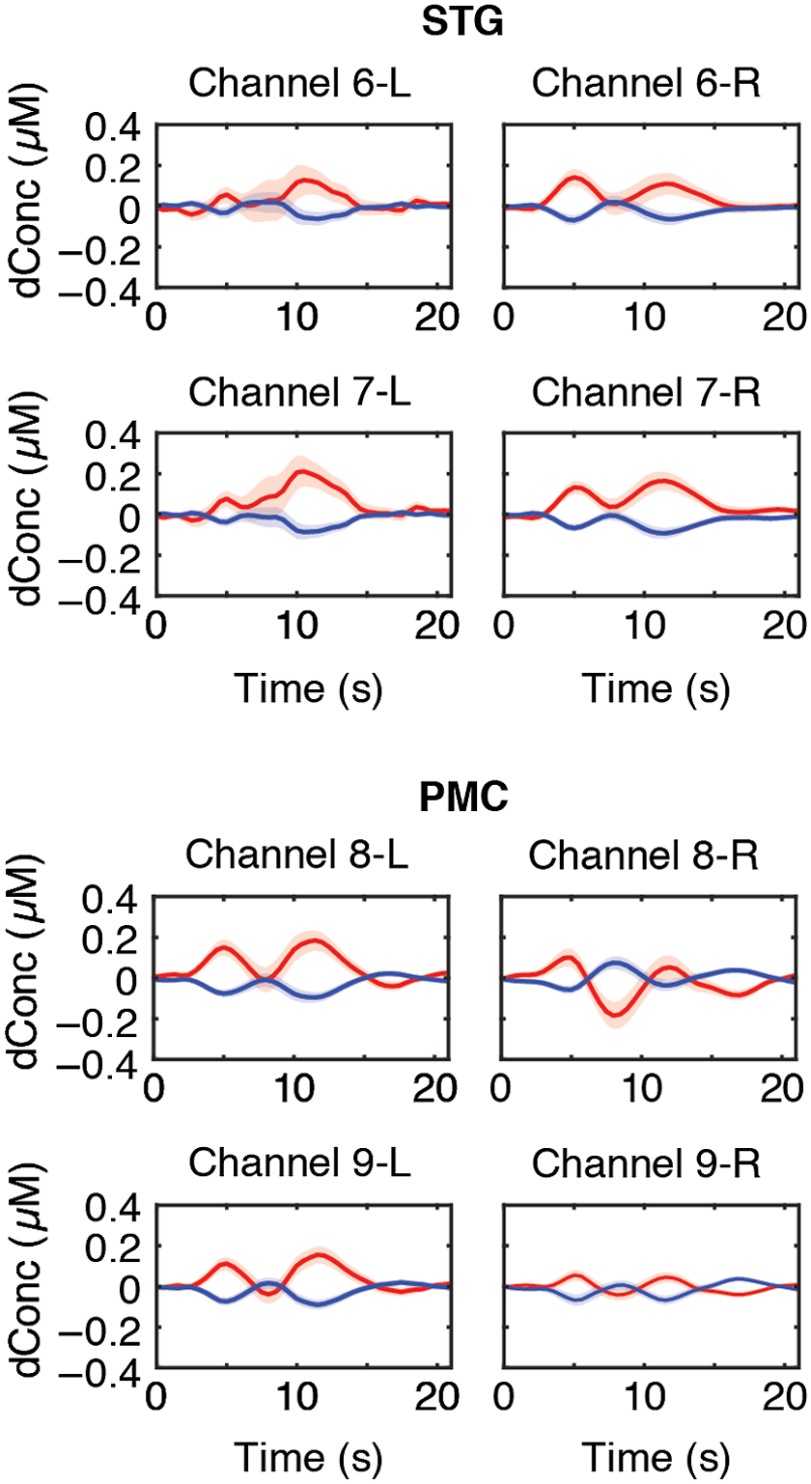
Grand averaged HbO (red) and HbR (blue) HRs with standard error curves for each channel are plotted by left (L) or right (R) ROI for the STG and PMC. Time 0 indicates trial onset when pictures were presented.

Figures 2 and 3 show the grand averaged HbO and HbR waveforms by ROI revealing the amplitude and time course of HbO (red traces) and HbR (blue traces) over all 30 experimental trials. Time 0 indicates when picture scenes were presented. The window of analysis was 3 to 8 s. The grand averaged HR data shows canonical HRs during the speech planning and production over speech motor regions. HbO and HbR concentration amplitudes reached an initial peak ∼4 to 6 s after stimuli onset.

**Fig. 4:**
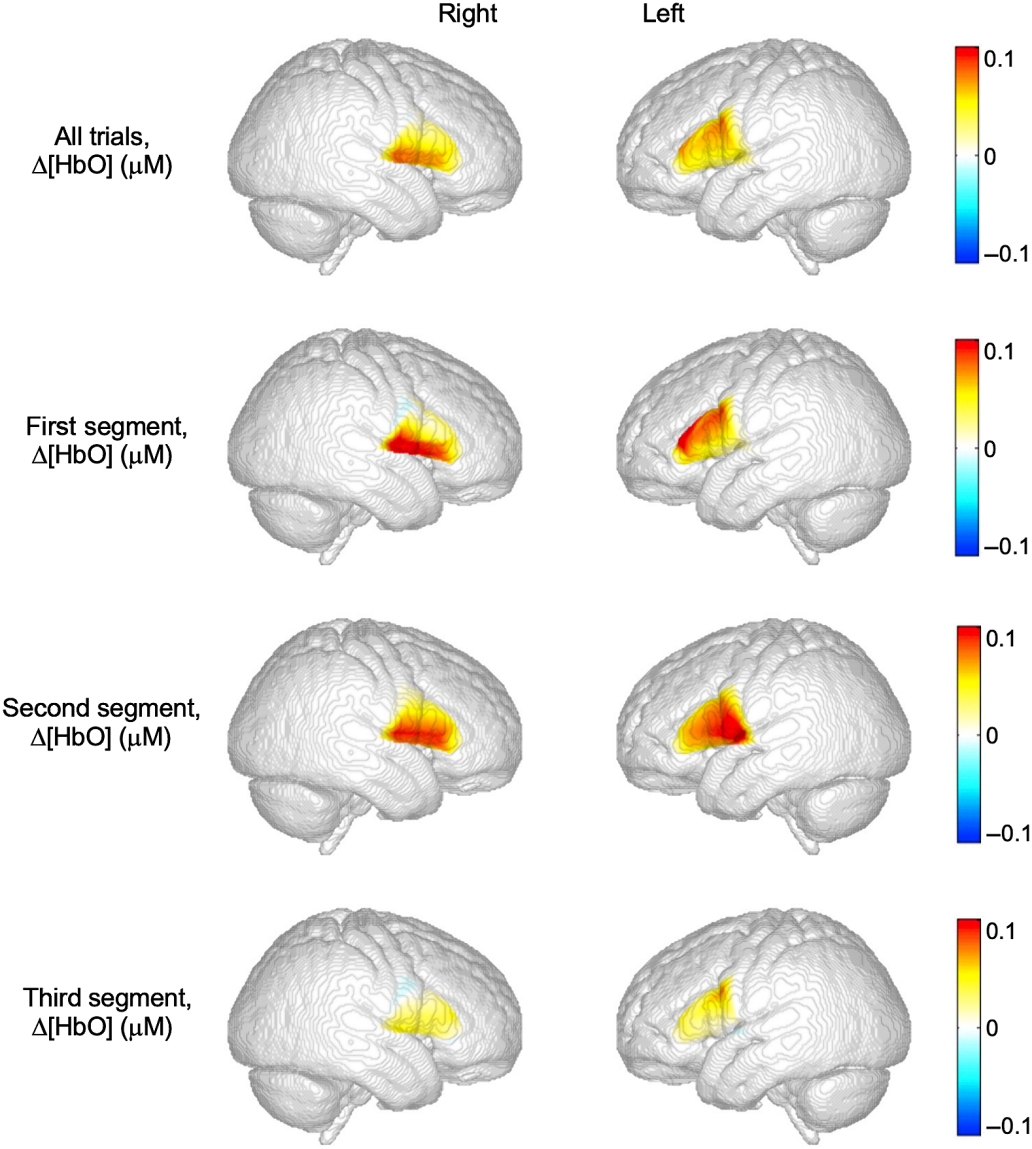
Topographic images of average changes in HbO response amplitude within the probe boundary by hemisphere and time segment. The top row shows HbO averaged across all experimental trials for the right and left hemispheres, while the other images show data over each respective time segment.

**Fig. 5:**
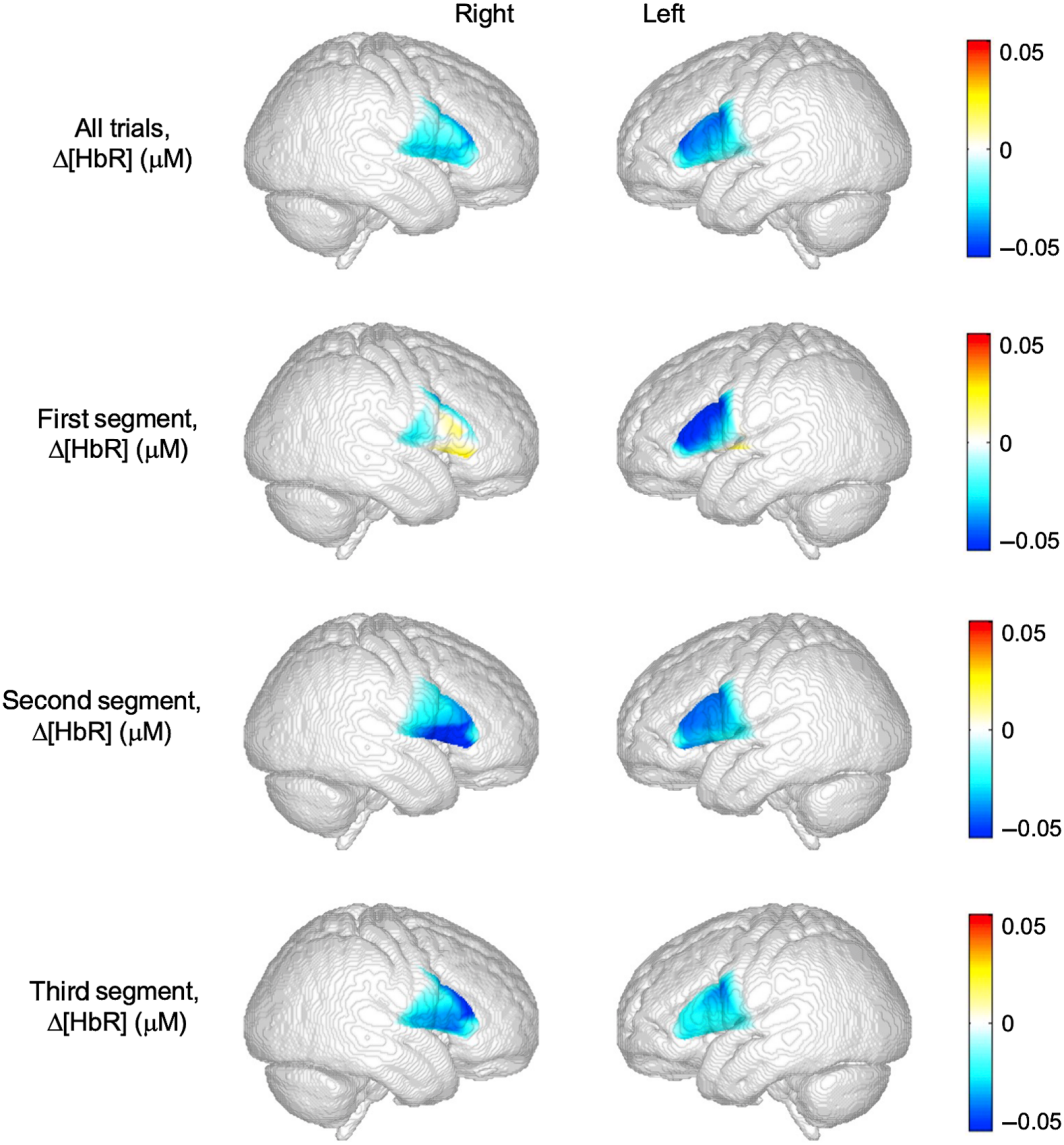
Topographic images of average changes in HbR response amplitude within the probe boundary by hemisphere and time segment. The top row shows HbR averaged across all experimental trials for the right and left hemispheres, while the other images show data over each respective time segment.

Figures 4 and 5 show topographic images of average HbO and HbR concentrations within the probe boundaries associated with spontaneous speech production within the 3 to 8 s post-stimulus analysis window. Channel-wise HbO and HbR concentrations were used to generate activation maps for the talk trials. The top row shows HbO and HbR concentration averaged across all experimental trials for the right and left hemispheres. The second, third, and fourth row show average HbO and HbR for the first, second, and third trials, respectively.

**Fig. 6:**
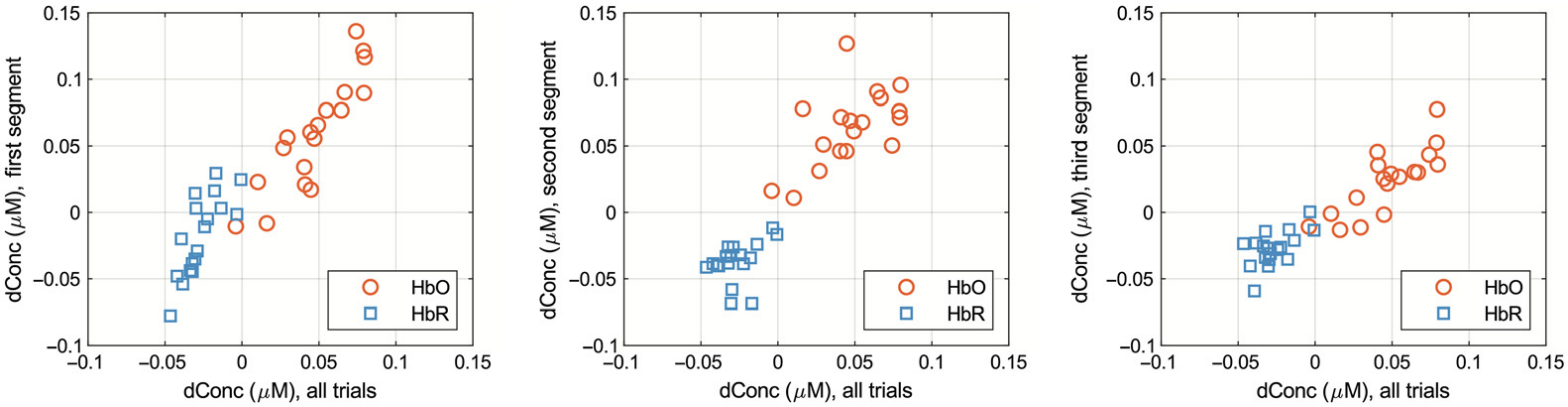
The correlations of average channel-wise HbO and HbR activations from all the 30 trials (x-axis) plotted against average channel-wise HbO and HbR activations from the first, second, and third segments. HbO is plotted in red and HbR in blue.

Correlations between average channel-wise HbO and HbR concentration changes within the window of analysis for all 30 trials and HbR and HbO averages for the early, middle, and late segments were computed to assess the strength of the relationship between these measures (Fig. 6). The correlation between averages for the first segment and the overall average was very strong for HbO (R=0.89, p<0.00) and strong for HbR (R=0.78, p<0.01). The correlation between the overall 30 trials and the second segment was (HbO: R=0.59, p=0.01; HbR: R=0.39, p=0.11), indicating a moderate correlation for HbO and a weak correlation for HbR. Finally, the correlation between the overall 30 trials and the third segment was (HbO: R=0.82, p<0.00; HbR: R=0.60, p=0.01), indicating a very strong correlation for HbO and strong correlation for HbR. On average, the highest HbO and HbR concentrations were seen during the second segment trials, yet correlational analysis revealed consistent HRs within the 3 to 8 s post-stimulus window for the early, middle, and late experimental trials.

There was not a significant difference in vIFG HbD response amplitudes across the three time segments, nor was the effect of hemisphere, or the interaction between hemisphere and segment significant [all ps 0.44 to 0.85]. We detected a significant segment × hemisphere interaction for dIFG activation F(2,72)=4.90, p=0.01. Bonferroni-corrected post hoc *t*-test comparisons indicated a significant difference between left and right dIFG HbD for the first segment t(36)=2.95, p=0.006; but not for the second t(36)=0.08, p=0.93 or third t(36)=−0.45, p=0.65 segments. We did not detect segment or hemisphere effects or interactions in PMC HbD response amplitudes [all ps 0.35 to 0.70] or STG HbD response amplitudes [all ps 0.44 to 0.85]. The ANOVA results for dIFG can be found in Table 1.

**Table 1:** Mean, standard error, and 95% confidence intervals for time segment by hemisphere ANOVA results for HbD response amplitudes for dIFG.

Segment	Hemisphere	Mean	Std. error	95% Confidence interval	
				Lower bound	Upper bound
1	Left dIFG	0.172	0.047	0.076	0.268
	Right dIFG	0.029	0.065	−0.103	0.162
2	Left dIFG	0.1	0.051	−0.004	0.204
	Right dIFG	0.096	0.06	−0.025	0.218
3	Left dIFG	0.059	0.054	−0.051	0.168
	Right dIFG	0.088	0.08	−0.075	0.251

## 4 Discussion

Using fNIRS in this study we assessed the common assumption held by neuroimaging investigations of speech production that participants engage speech and language networks in a consistent manner (i.e., they demonstrate stable HRs) across multiple experimental trials. We explored the consistency of HRs in speech and language regions during a picture description task in typically developing children. Results indicated that HbO and HbR across channels in early, middle, and late segments correlated with HbO and HbR calculated across the entire experiment to different degrees. Correlations for HbO tended to be stronger than correlations for HbR. Correlations between overall HbO and HbR and the first and last segments were the strongest, while correlation coefficients decreased between overall HbO and HbR in the second segment (weak to moderate in strength). This pattern of findings reveals an overall high degree of consistency of HbO and HbR across all channels between segments and the overall experiment.

ANOVA analysis explored the consistency of HRs within a particular ROI. We found that average HbD concentrations of the most left and right hemisphere speech and language ROIs did not significantly change over the course of the experiment—the early, middle, and late segments. Behaviorally, children completed the picture description task in a consistent manner over the course of the experiment. Taken together, findings revealed that HRs over speech and language regions can be reliably measured during relatively short paradigms with typically developing school-aged children. The exception to this consistent pattern was the average HbD concentration recorded over dIFG in the left hemisphere. A hemisphere by segment interaction indicated that dIFG activation in the left hemisphere was significantly greater than right hemisphere activation for early trials. Left IFG is a critical region for speech production.^9^ Our findings indicated that this region was engaged to a greater degree than the right homologous areas of the earlier trials of the task.

The implications of HRs changing across an experimental paradigm may be profound depending on the field and population under investigation. For example and with data from this study, the left and right IFG are integral components of speech production networks.^33^ The left IFG, in particular, is hypothesized to comprise a critical link between phonology and speech-motor control.^7^^,^^8^ Speech sound motor programs are acquired and refined during development and are hypothesized to be stored in left IFG for assembly and ultimate speech execution [see, Ref. 33 for discussion of speech sound and feedback control maps]. In our field of stuttering research, our work and others have shown that left hemisphere motor areas are often underactivated and right hemisphere motor regions are often overactivated in people who stutter compared to those who do not stutter,^16^^,^^17^^,^^44^^–^^47^ supporting the view that speech motor internal models are not as robust in people who stutter.^48^^,^^49^ Data from the present study suggest that this laterality difference in speech motor regions of people who stutter may be further specified by considering the stability of HRs over an experimental paradigm. Specifically, it is possible that these laterality effects may change over the course of an experiment in particular time segments or ROIs, as evidenced by the laterality difference found in this study regarding left IFG activation of children with typical development. Such considerations may lead to a deeper understanding of speech motor control network functioning in both disordered and neurotypical populations.

It is possible that this difference between activation of left dIFG and right dIFG during the early segment was mediated by differences in linguistic content of the utterances children produced. In a spontaneous speaking task such as picture description, the specific linguistic content (i.e., the semantic, morphological, and syntactic complexity) is not controlled across subjects and trials. However, this possibility is unlikely given that children completed the task in a consistent manner producing utterances of similar length across the time segments. It could also be that the HR over left dIFG decreased in the later trials as children became more efficient with the speech production task. Established models of speech production and formulation hypothesize that activity in regions of speech-language networks will change in activation as speech networks operate more efficiently.^7^^,^^9^ Regardless of the cause, our findings suggest that the number of trials in this paradigm could have been reduced without affecting the overall pattern of results in this study because the significant hemisphere by ROI interaction was found in the first segment only.

It is important to note that the children in the study were typically developing. Thus, the findings may not extrapolate to children with speech and language disorders, as these populations may show distinctly different HRs over the course of the experiment reflective of processes related to their specific disorder. Relatedly, HR consistency may change throughout development. We did not explore age effects on HRs in the current study, although our previous study with children in this age range did not support a significant relationship between age and HbO amplitudes.^16^ Finally, we did not record short channels or other measures of systemic physiology. Thus, our hemoglobin data may have been influenced by noise from extracerebral sources. Future research should incorporate these measures that allow the separation of systemic contribution to neural responses.

In sum, researchers should critically evaluate and pilot their paradigms to assess the consistency of HRs over ROIs before averaging across experimental trials to optimize their experiments. Habituation or other uncontrolled effects could potentially mask patterns of activation related to the task. This is an important step that can increase the efficiency of paradigms and the translatability of findings.
